# Therapeutic effect and potential mechanism of Fufang Danshen dripping pills for stable coronary heart disease: a randomized controlled trial

**DOI:** 10.3389/fcvm.2025.1506917

**Published:** 2025-01-29

**Authors:** Li-qin Meng, Pei-ying Huang, Qing-min Li, Yu-chao Feng, Ding-jian Li, Guang-long Wu, Bo-wen Ao, Guo-chao Wu, Guo-xiong Zhang, Bo-jun Chen

**Affiliations:** ^1^ The Second Clinical Medical College, Guangzhou University of Chinese Medicine, Guangzhou, China; ^2^ Department of Geriatrics, Yangjiang People's Hospital, Yangjiang, China; ^3^ Emergency Department, Guangdong Provincial Hospital of Traditional Chinese Medicine, Guangzhou, China; ^4^ Department of Chinese Medicine, First People's Hospital of Chenzhou City, Chenzhou, China; ^5^ Guangdong Provincial Key Laboratory of Research on Emergency in Traditional Chinese Medicine, Clinical Research Team of Prevention and Treatment of Cardiac Emergencies with Traditional Chinese Medicine, Guangzhou, China; ^6^ Department of Laboratory, Yangjiang People's Hospital, Yangjiang, China; ^7^ Department of Cardiology, Yangjiang People's Hospital, Yangjiang, China; ^8^ The First Clinical Medical College, Guangdong Medical University, Zhanjiang, China; ^9^ Physical Examination Department, Guangdong Provincial Hospital of Traditional Chinese Medicine, Guangzhou, China

**Keywords:** Fufang Danshen dripping pills, stable coronary heart disease, triglycerides, angina frequency, lipidomics

## Abstract

**Background:**

The efficacy and mechanism of Fufang Danshen dripping pills (FFDS) in the secondary prevention of stable coronary heart disease (SCHD) is currently undetermined. This study aims to investigate the efficacy and preliminary mechanism by which FFDS may impact the progression of SCHD.

**Methods:**

Based on randomization, we administered oral FFDS to 30 patients with SCHD in addition to conventional treatment for 30 days. After treatment, three-months major adverse cardiovascular events (MACE) were assessed as the primary outcome. Additionally, we evaluated the patients' Seattle Angina Questionnaire score, blood pressure, circulating levels of total cholesterol, low-density lipoprotein cholesterol, high-density lipoprotein cholesterol, triglycerides, C-reactive protein, platelets, alanine aminotransferase, aspartate aminotransferase, serum creatinine, and fasting blood glucose as the secondary outcomes. Furthermore, we utilized mass spectrometry analysis, network pharmacology, and lipidomics to predict the potential mechanisms of FFDS in the treatment of SCHD.

**Results:**

Following treatment, FFDS demonstrated significant improvements in serum triglyceride levels (*P* = 0.013) and a reduction in the frequency of angina episodes (*P* = 0.021). We conducted mass spectrometry analysis on FFDS and identified 236 chemical components. Lipidomics further confirmed triglycerides as key lipids affected by FFDS. By integrating these findings with network pharmacology targets, we highlighted the potential roles of LPL, CD36, FABPpm, L-FABP, LCAT, and CEPT in fat digestion, absorption, and metabolism pathways, suggesting their involvement in FFDS's treatment of SCHD by reducing triglycerides.

**Conclusion:**

In individuals with SCHD, the administration of FFDS has been shown to effectively reduce circulating triglyceride levels and decrease the frequency of angina episodes. This therapeutic effect is likely due to the active components of FFDS targeting key proteins: LPL, CD36, FABPpm, L-FABP, LCAT, and CEPT.

**Clinical Trial Registration:**

https://www.chictr.org.cn/, identifier (ChiCTR2400080149).

## Introduction

Coronary heart disease (CHD) represents a significant global health burden, with high morbidity and mortality (1). Stable coronary heart disease (SCHD) is a significant subtype of CHD, encompassing conditions such as silent myocardial ischemia, stable angina pectoris, and ischemic cardiomyopathy (1–3). In the context of SCHD, the term “stable” should not be misconstrued as indicating minimal risk or negligible impact, thereby diminishing the necessity for public awareness. On the contrary, SCHD is influenced by a multitude of persistent risk factors that continue to affect individuals over time (2). The progression of the disease, driven by these factors, remains unpredictable, and patients are at a continual risk of experiencing major adverse cardiovascular events (MACE) at any given time (2).

Secondary prevention is a mainstream approach to reduce the occurrence of adverse events in SCHD. These protocols generally include lifestyle modifications, such as dietary adjustments and regular physical activity, the administration of optimal medical therapies (e.g., statins, beta-blockers, and ACE inhibitors), revascularization procedures such as percutaneous coronary intervention or coronary artery bypass grafting, and antiplatelet therapy to mitigate thrombotic complications (4). However, recent evidence underscores that patients with SCHD continue to face a substantial risk MACE despite adherence to contemporary secondary prevention protocols (5, 6). Research indicates that despite adherence to secondary prevention strategies, the risk of MACE remains significant, with a 5-year probability reaching up to 35% (7).

In this context, there remains a need for further optimization in the treatment of patients with SCHD. Recent advancements have introduced additional therapeutic strategies, including profound lipid-lowering and inflammation-modifying agents, as well as novel antithrombotic combinations (2). However, it is important to acknowledge that while these newly implemented measures have the potential to reduce the risk of MACE, they also present notable limitations. For instance, a “residual risk” persists even after significant reductions in low-density lipoprotein cholesterol (LDL-C) (8). Additionally, the long-term use of inflammation-modulating drugs may elevate the risk of infection, and newer antithrombotic treatments are associated with an increased risk of bleeding (9, 10). Consequently, the pursuit of novel therapeutic agents to address the risks associated with SCHD remains an ongoing imperative.

Fufang Danshen dripping pills (FFDS) represent an oral pharmaceutical formulation developed through advanced drug processing techniques to extract the active constituents of Danshen (*Salviae Miltiorrhizae Radix et Rhizoma*), borneol (*Borneolum Syntheticum*), and notoginseng (*Notoginseng Radix et Rhizoma*). Previous research has indicated that FFDS may offer therapeutic benefits in the management of hyperlipidemia, myocardial ischemia, and angina pectoris associated with CHD in clinical settings, as well as in the treatment of hyperlipidemia and myocardial ischemia in animal models (11–13). However, its potential clinical efficacy in improving SCHD has not been systematically investigated. Furthermore, as FFDS is an herbal compound, its associated molecular mechanisms in relation to the disease require urgent elucidation. To evaluate the effectiveness of FFDS in patients with SCHD and to partially elucidate its mechanism of action, a comprehensive study was undertaken, combining a randomized controlled trial (RCT) with network pharmacology and lipidomics analysis.

## Method

### Inclusion criteria

1. The study population comprised patients aged 18 years and older, recruited from Yangjiang People's Hospital.
2. The patient's prior coronary angiography revealed stenosis of greater than or equal to 50% in the internal diameter of the main left or right coronary artery, or its primary branches, irrespective of any previous coronary interventions, such as percutaneous coronary intervention (PCI). Upon enrollment in the trial, patients may exhibit stable angina or present as asymptomatic myocardial ischemia or asymptomatic old myocardial infarction (14).
3. Each patient gave informed consent and signed a written informed consent.

### Exclusion criteria

1. The patient's coronary angiography revealed complete occlusion of the coronary arteries.
2. Patient experienced an acute myocardial infarction or unstable angina within the past three months.
3. Patient presented with a new onset of Left Bundle Branch Block.
4. Patient used steroids or immunosuppressants within one month prior to enrollment.
5. Patient had other underlying conditions that could affect outcome assessment, such as cancer, sepsis, autoimmune diseases, hyperthyroidism, or mental illness.
6. Patient exhibited organ dysfunction.
7. Patient was pregnant or lactating.

### Intervention

Patients in the control group received standard secondary prevention therapy according to the guideline (15), which included the administration of lipid-lowering drugs, nitrates, aspirin and/or clopidogrel, β-receptor blockers, calcium channel blockers, and renin-angiotensin-aldosterone system inhibitors. In the experimental group, patients received FFDS (Z10950111) orally, taking 10 pills three times a day with 30 days as the treatment course, in addition to the standard secondary prevention therapy described above.

### Baseline data

Upon enrollment, comprehensive baseline data were collected for each patient, encompassing gender, age, body mass index (BMI), blood pressure, heart rate, fasting blood glucose, blood lipids, platelet count, underlying CHD-related diseases, C-reactive protein (CRP), and Seattle Angina Questionnaire (SAQ) scale (The SAQ scale encompasses 5 dimensions, comprising a total of 19 items. These dimensions are Physical Limitation, Stability of Angina, Angina Frequency, Satisfaction with Treatment, and Disease Knowledge). Subsequently, patients received treatment in accordance with the predetermined protocol.

### Primary outcome

Following a 3-month period subsequent to the 30-day trial cycle, the incidence of MACE was documented and considered the primary outcome.

### Secondary outcomes

Post the 30-day trial cycle, blood pressure and SAQ scores were reassessed. At the same time, blood samples were collected from surviving patients for laboratory analysis. This analysis included the measurement of fasting glucose, high-density lipoprotein cholesterol (HDL), low-density lipoprotein cholesterol (LDL), total cholesterol (TC), triglycerides (TG), CRP, platelet count, alanine aminotransferase (ALT), aspartate aminotransferase (AST), and serum creatinine levels. All the above-mentioned indicators were designated as the secondary outcomes.

### Study style

Eligible patients were randomly assigned using a random number sequence enclosed within opaque envelopes. This study employed an open-label, non-blinded RCT design.

### Sample size

Based on previous research (16), we determined the necessary sample size for an intervention to effectively treat CHD. Using a two-sided test with a significance level of 0.05 and a test power of 90%, we calculated a total sample size of *N* = 48, divided into 2 groups of 24 cases each. Factoring in a 20% estimated dropout rate, the minimum sample size for the study was adjusted to *N* = 60, which means that each group requires 30 participants.

### Ethics review

The clinical study component has received ethical approval from the Ethics Committee of Yangjiang People's Hospital on May 7th, 2020. This study was registered in https://www.chictr.org.cn/ (ChiCTR2400080149) and we followed the CONSORT guidelines with the CONSORT checklist attached as the attachment.

### Collection of targets for FFDS in the treatment of SCHD

We precisely measure approximately 1.0 g of chopped FFDS and place it into an Erlenmeyer flask. The FFDS utilized in this experiment was obtained from the identical batch of medication (Z10950111) as that administered to the patients. To prepare the mixed sample, five samples, each weighing 0.2 g, were randomly selected from this batch and subsequently combined. Subsequently, we add 5 ml of sterile water and agitate the flask until the FFDS is fully dissolved. Ultrasonic extraction is then conducted at room temperature for 30 min. Following this procedure, the mixture is centrifuged at 12,000 r/min for 10 min, after which the supernatant is collected for analysis (ultrapure water were used as control). The analysis is performed using a Q Exactive mass spectrometer (Thermo Fisher), and the resulting data is queried against a database to ascertain the composition of FFDS. Finally, the TCMSP database (https://www.tcmsp-e.com/#/home) is utilized to predict the targets of each identified chemical component of FFDS (17).

Since dyslipidemia is one of the main risk factors for MACE, previous study has also suggested that FFDS has a certain therapeutic effect on hyperlipidemia (11). Consequently, this study emphasizes the role of FFDS in addressing hyperlipidemia within the context of treating SCHD. To comprehensively identify potential targets for FFDS in the treatment of SCHD, we incorporated hyperlipidemia-related targets into our analysis. The GeneCards disease database (https://www.genecards.org/) was queried using “coronary heart disease” and “hyperlipidemia” as keywords to identify disease-related targets, and the common targets between the two diseases were collected. Screening parameters were set in the Uniprot database (https://www.uniprot.org/), with human species selection, “Reviewed Swiss-Prot” as the verified protein target, and protein gene names standardized. Finally, by intersecting the action targets of FFDS with the disease targets, potential targets through which FFDS may exert its effects for SCHD were identified.

### KEGG enrichment analysis

For the KEGG enrichment analysis of the identified targets, we employed the “org.Hs.eg.db” and “clusterProfiler” packages within the R programming environment. The analysis results were subsequently visualized using the “ggplot2” package. KEGG pathways with an adjusted *P*-value of less than 0.05 were deemed statistically significant.

### Lipidomic analysis of patient blood

In order to further explore the mechanism of FFDS affecting blood lipids, a lipidomic analysis was conducted using venous blood samples collected from each patient (30 patients in FFDS group and 30 patients in control group) at the end of the 30-day treatment course. The analysis procedure involved the following steps:

Sample preparation: 100 μl of serum was mixed with 1.5 ml of chloroform/methanol (V:V = 2:1) and 0.5 ml of pure water. The mixture was vortexed for 1 min and centrifuged at 3,000 rpm for 10 min. The organic phase was transferred to a clean glass tube and centrifuged again at 2000rpm for 10 min. The organic phase was then concentrated, dried, and reconstituted with isopropanol/methanol (V:V = 1:1) for subsequent LC-MS detection and analysis.
LC-MS detection: The analysis utilized a C18 chromatographic column (Kinetex C18, 100 mm × 2.1 mm, 1.9 μm). The column temperature was set at 45°C, and the flow rate was maintained at 0.4 ml/min. The mobile phase consisted of two components: A (acetonitrile:water, V:V = 60:40) and B (acetonitrile:isopropanol, V:V = 10:90). A gradient elution program was employed as follows: 0–2 min, 70% A and 30% B; 2–20 min, 70% A and 30% B; 20–40 min, 100% B; 40–45 min, 70% A and 30% B. The injection volume was 4 μl, and the autosampler temperature was maintained at 4°C. Mass spectrometry parameters were set differently for positive mode (ES+) and negative mode (ES-).

Lipid Search software was used to extract and normalize the data obtained from LC-MS analysis in both positive and negative modes. The software provided information such as lipid ion (Lipidlon), classification (Class), molecular weight (CalcMz), ion molecular formula (IonFormula), and peak intensity. The data was then imported into SIMCA-P13.0 software for principal component analysis (PCA) and partial least squares-discriminant analysis (PLS-DA). Model sorting and verification were performed to identify any potential overfitting. Qualitative analysis was conducted using Lipid Search software, and differential metabolite information was obtained by combining the variable importance for projection (VIP) value (VIP ≥ 1) and the *P*-value from the *t*-test (*P* < 0.05) for intergroup comparisons. The identified lipid metabolites were cross-referenced with the Human Metabolome Database (HMDB) and LIPID MAPS Structure Database (LMSD) for further validation, and KEGG database was utilized for metabolite analysis and pathway prediction (adjusted *P* < 0.05).

We integrated the results of the target-related KEGG analysis with those of the differential lipid KEGG analysis to elucidate the pathways through which FFDS influences CHD and lipid disorders. Subsequently, all targets and identified pathways were submitted to the KEGG official website (https://www.genome.jp/kegg/kegg_ja.html) to ascertain the primary targets and pathways involved in the function of FFDS, as visualized through specific mechanistic maps.

### Statistical analysis

We assessed the normality of continuous variables using the *Shapiro-Wilk* test and employed appropriate statistical tests for data analysis. For variables that followed a normal distribution, we utilized the independent sample *t*-test and presented the results as *mean* *±* *standard deviation*. For variables that did not conform to a normal distribution, we used the *Mann-Whitney U*-test and reported the data as median (*25th quantile, median, 75th quantile*). Categorical variables were compared between the two groups using the *chi-square* test. A significant level of *P* < 0.05 was considered statistically significant. Analyses were performed using SPSS 19.0.

## Results

### Baseline characteristics

The treatment group and the control group included 30 patients respectively. No patients withdrew from the study during the treatment period. The demographic characteristics and baseline parameters of the patients, including age, gender, comorbidities (hypertension and diabetes), smoking history, BMI, blood pressure, heart rate, CRP, fasting blood glucose, platelet count, LDL, HDL, TG, TC, and SAQ score were compared between the groups. Statistical analysis revealed no significant differences in these variables between the groups (*P* > 0.05), indicating comparable baseline characteristics (Table 1).

**Table 1:** Baseline characteristics of patients in the two groups.

	FFDS group (30)	Control group (30)	*P*-value
Age (years)	(59.75, 67.00, 73.00)	(65.25, 72.00, 76.25)	0.069
Sex (male)	25 (83.3%)	19 (63.3%)	0.143
BMI	23.41 ± 2.87	23.32 ± 2.83	0.906
Systolic blood pressure	141.83 ± 21.68	139.13 ± 21.86	0.633
Diastolic blood pressure	84.57 ± 2.32	79.50 ± 2.29	0.125
Heart rate	76.03 ± 10.87	73.80 ± 15.60	0.523
Basic CRP	(2.13, 4.30, 12.85)	(1.73, 3.00, 10.53)	0.359
Basic fasting glucose	(5.09, 6.41, 8.83)	(5.03, 6.21, 7.23)	0.446
Basic platelet count	(202.50, 228.50, 259.00)	(183.00, 229.50, 252.25)	0.564
Basic low-density lipoprotein cholesterol	(2.87, 3.35, 4.40)	(2.01, 3.00, 4.31)	0.261
Basic high-density lipoprotein cholesterol	(0.98, 1.18, 1.46)	(0.98, 1.15, 1.34)	0.971
Basic total cholesterol	5.30 ± 1.13	4.99 ± 1.48	0.362
Basic triglyceride	(1.02, 1.46, 1.97)	(0.76, 1.28, 1.76)	0.337
Smoking	13 (43.30%)	9 (30.00%)	0.422
Hypertension	11 (36.70%)	11 (36.70%)	1.000
Diabetes	1 (3.30%)	5 (16.67%)	0.195
Seattle angina questionnaire			
Physical limitation	53.62 ± 13.06	57.16 ± 15.02	0.334
Angina stability	54.39 ± 14.09	49.74 ± 13.71	0.200
Angina frequency	53.89 ± 14.65	58.12 ± 13.46	0.248
Treatment satisfaction	49.00 ± 14.02	49.42 ± 14.05	0.906
Disease knowledge	52.09 ± 13.84	52.87 ± 14.99	0.835

### Clinical outcomes

Statistical analysis revealed that the addition of oral FFDS to conventional Western medicine treatment effectively reduced the serum TG levels and angina frequency (*P* < 0.05) and may reduce the patient's 3-month MACE incidence (*P* = 0.080) (One patient from the FFDS group and five patients from the control group were admitted to hospital due to myocardial infarction and received percutaneous coronary intervention) (Table 2). No statistically significant differences were observed between the FFDS group and the control group for other measured indicators.

**Table 2:** Outcomes comparison of the two groups of patients.

	FFDS group (30)	Control group (30)	*P*-value
Primary outcome			
Major adverse cardiovascular events	1 (3.30%)	5 (16.70%)	0.080
Secondary outcome			
AST	(19.20, 23.70, 27.70)	(17.08, 20.70, 25.63)	0.220
ALT	(20.48, 27.50, 36.10)	(17.33, 23.65, 31.88)	0.225
Serum creatinine	84.23 ± 15.54	88.67 ± 23.33	0.390
CRP	(1.10, 2.03, 3.60)	(1.47, 2.25, 3.28)	0.615
Systolic blood pressure	130.73 ± 11.92	132.60 ± 13.08	0.566
Diastolic blood pressure	88.1 ± 7.64	87.17 ± 8.34	0.653
Fasting glucose	(5.23, 5.81, 6.65)	(5.45, 6.09, 6.54)	0.225
Platelet count	(205.75, 226.00, 259.00)	(183.50, 231.50, 255.25)	0.530
Low-density lipoprotein cholesterol	(1.39, 1.75, 2.37)	(1.56, 1.91, 2.72)	0.318
High-density lipoprotein cholesterol	(0.97, 1.20, 1.54)	(1.05, 1.28, 1.49)	0.336
Total cholesterol	(3.14, 3.58, 4.20)	(3.13, 3.59, 4.36)	0.859
Triglyceride	1.08 ± 0.41	1.44 ± 0.64	0.013
Seattle angina questionnaire			
Physical limitation	64.91 ± 11.52	63.08 ± 13.47	0.574
Angina stability	59.41 ± 15.49	57.69 ± 13.78	0.651
Angina frequency	65.33 ± 15.10	56.41 ± 13.93	0.021
Treatment satisfaction	63.86 ± 11.77	60.09 ± 12.75	0.239
Disease knowledge	55.51 ± 13.05	58.48 ± 12.29	0.369

### Targets of FFDS in treating SCHD

**Figure 1:**
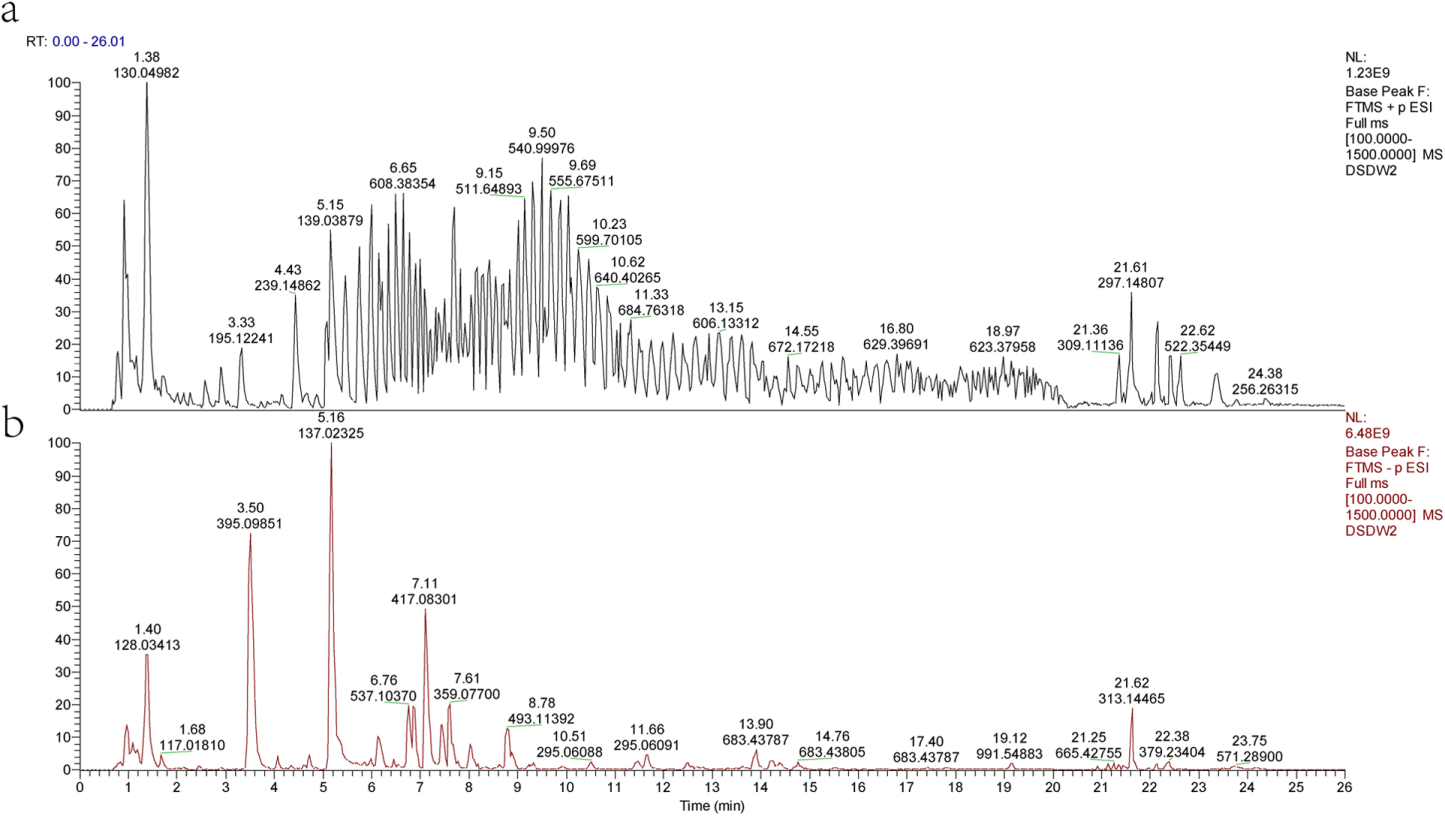
Mass spectrometry analysis results of FFDS. Results in cation mode **(a)** Results in anion mode **(b)**.

After Q Exactive mass spectrometer analysis and database comparison, we identified 236 chemical components contained in FFDS (Figure 1, Supplementary Table S1). Based on TCMSP database, we obtained 536 unique therapeutic targets that FFDS acts upon in the human body. Subsequently, we converted these targets into gene symbols, resulting in a total of 201 symbols (Supplementary Table S2).

**Figure 2:**
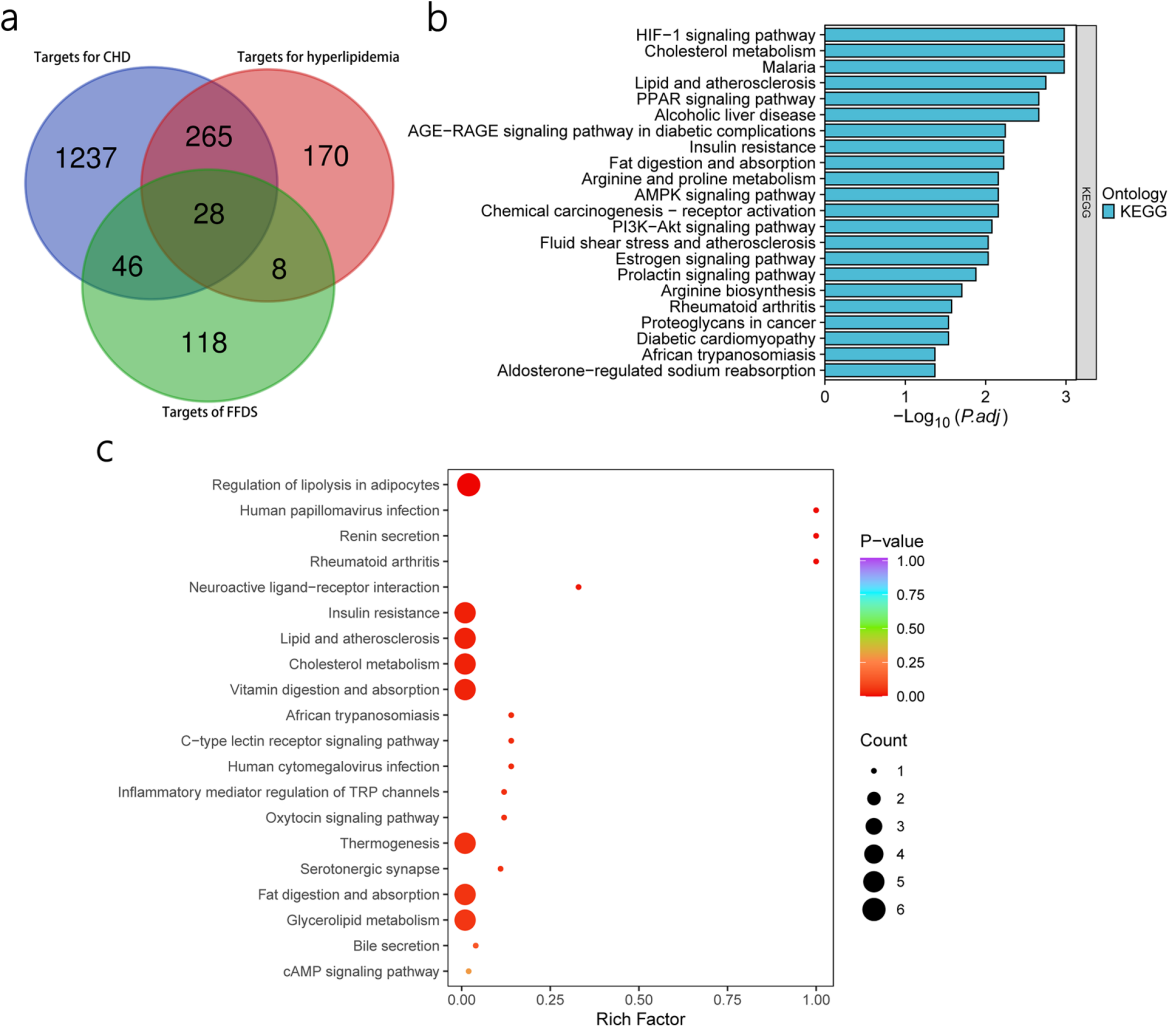
Prediction of FFDS targets in CHD and hyperlipidemia and KEGG enrichment analysis based on predicted targets and differential lipids. Prediction of the target of FFDS in CHD and hyperlipidemia **(a)** KEGG enrichment analysis based on predicted targets **(b)** KEGG enrichment analysis based on differential lipids **(c)**.

Through the GeneCards database, we compiled 1,576 therapeutic targets for CHD and 471 therapeutic targets for hyperlipidemia (Supplementary Table S3). Taking the intersection of these targets, we identified 293 targets that are regarded as SCHD targets. Finally, we overlapped the FFDS targets with the SCHD targets, leading to the prediction of 28 therapeutic targets that FFDS may act upon in patients with SCHD (Figure 2a, Supplementary Table S4).

### KEGG enrichment analysis of predicted targets

Through KEGG enrichment analysis of the 28 identified targets, we identified 22 significant pathways (Figure 2b).

### PCA multivariate statistical analysis and OPLS-Da analysis

**Figure 3:**
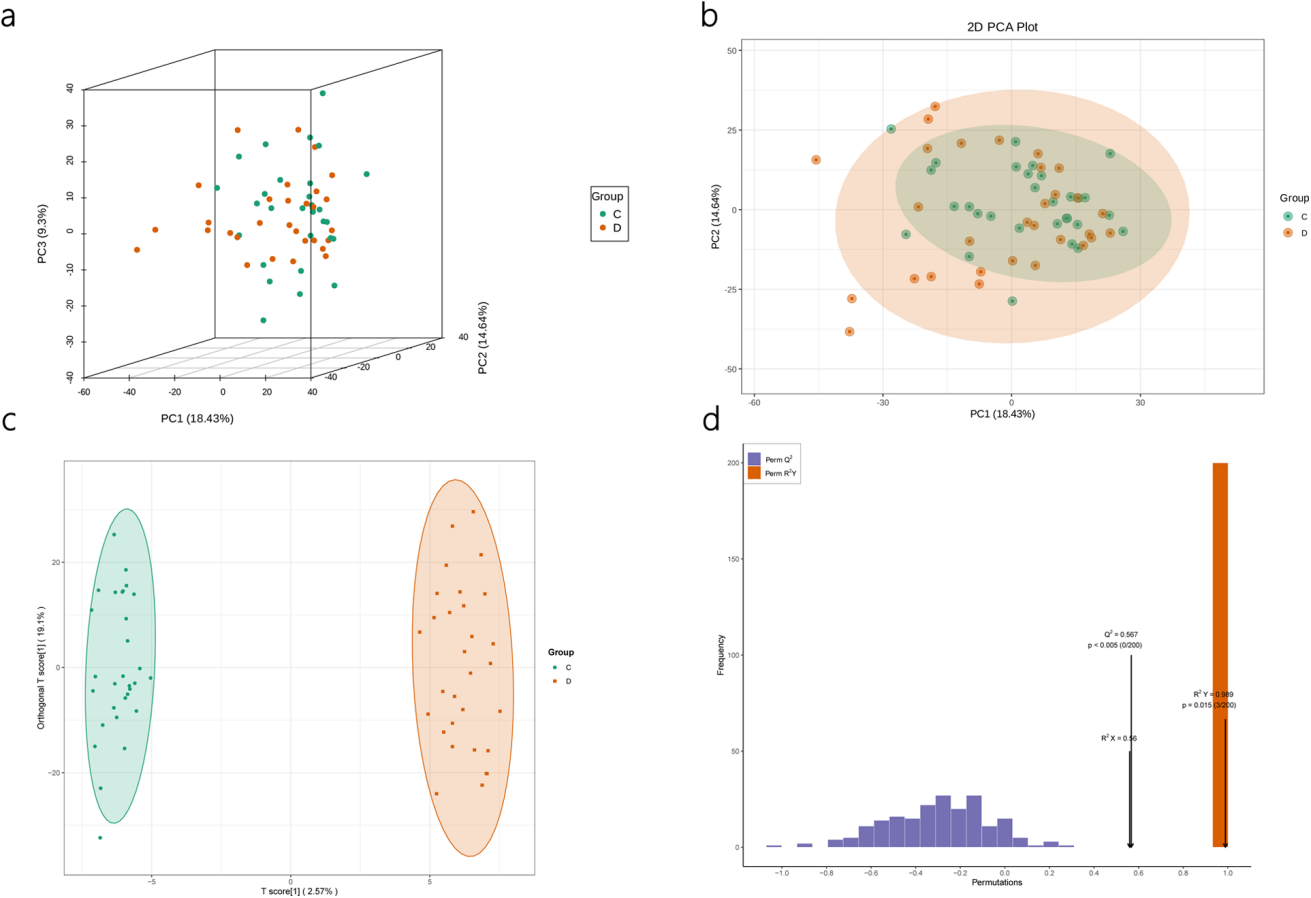
Quality assessment of lipidomics results. PCA results of lipidomics **(a,b)**. OPLS-DA Model **(c)** and Model Validation for Lipidomics **(d)**.

Prior to conducting the differential analysis, PCA was performed on the grouped samples to compare the differences and assess the variability within and between the groups. As shown in Figures 3a,b, the components of the FFDS group and the control group exhibit relatively proximity, with only a few discernible differences.

Subsequently, OPLS-DA was employed to elucidate the differences between the two groups and identify the corresponding differential metabolite results. The model parameters indicate the reliability of the model in explaining the intergroup differences, and the validation plot demonstrates the absence of “overfitting” phenomenon (Figures 3c,d).

### Differential lipid screening and KEGG pathway enrichment

Based on pre-defined criteria, three differential lipids, namely glycophosphatidylinositol, oxidized lipids, and TG, were identified. Additionally, KEGG enrichment analysis revealed 25 differential pathways associated with these lipids (Figure 2c). Notably, these identified pathways overlap with the KEGG pathways previously identified through network pharmacology, which were considered as the potential therapeutic pathways through which FFDS exerts its effects. Specifically, the overlapped pathways include Insulin resistance, Fat digestion and absorption, Cholesterol metabolism, and Lipid and atherosclerosis, highlighting their relevance in the therapeutic actions of FFDS on SCHD.

**Figure 4:**
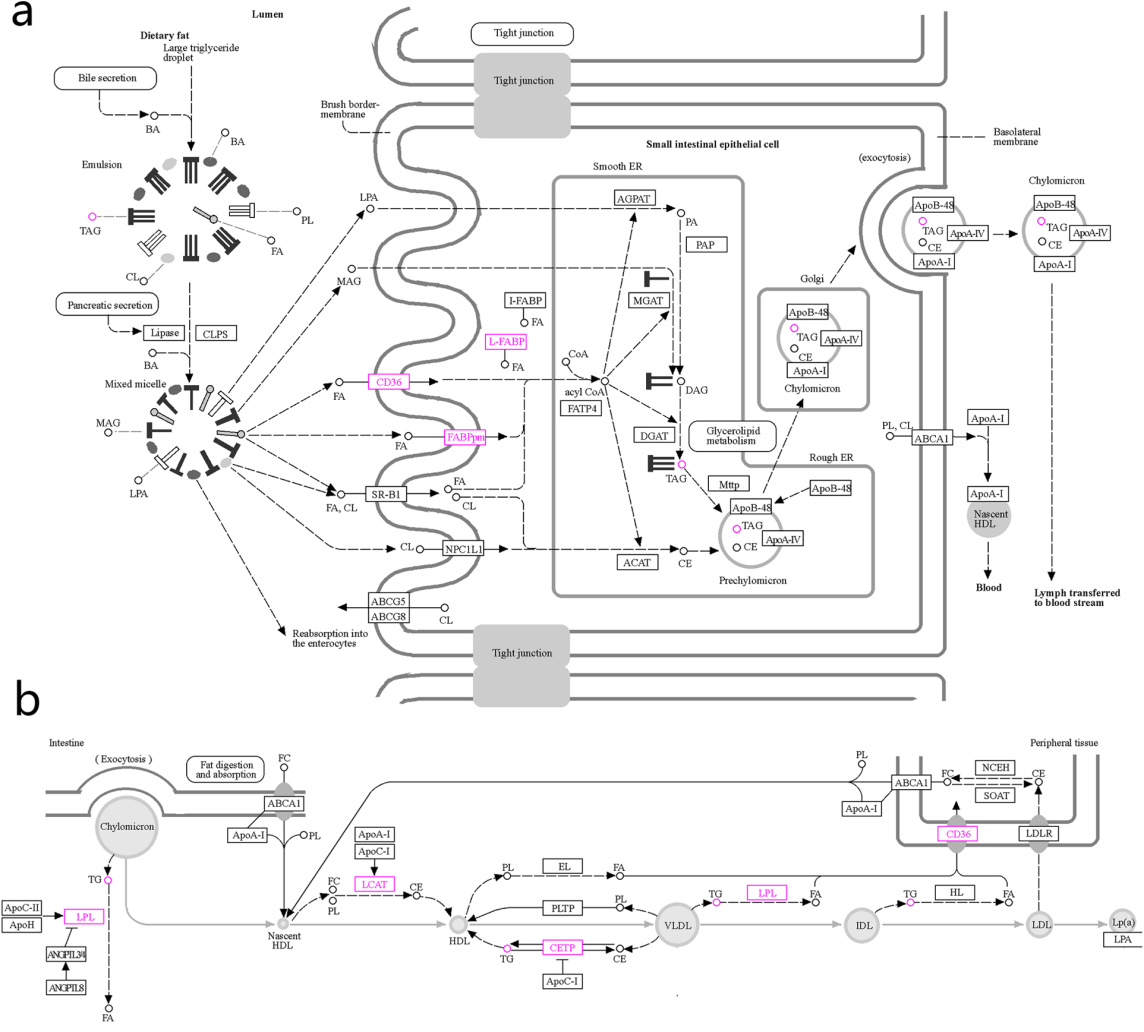
The possible mechanism of FFDS regulating TG and alleviating the progression of SCHD. Mechanisms by which FFDS regulate TG production **(a)** Mechanism of FFDS regulating TG production and mechanism of reducing the probability of dyslipidemia leading to SCHD **(b)** The highlighted icons represent specific targets that FFDS may interact with. CD36: platelet glycoprotein 4; FABPpm, aspartate aminotransferase; L-FABP, fatty acid binding protein; CETP, cholesteryl ester transfer protein; LPL, lipoprotein lipase; LCAT, lecithin-cholesterol acyltransferase; TG/TAG, triglyceride; FA, fatty acid.

We input the 28 targets of FFDS targeting SCHD and the 5 pathways jointly identified by network pharmacology and lipid metabolomics into the KEGG official website. The results highlight the action of Lipoprotein lipase (LPL), platelet glycoprotein 4 (CD36), aspartate aminotransferase (FABPpm), cytoplasmic fatty acid binding protein (L-FABP), lecithin-cholesterol acyltransferase (LCAT), and cholesteryl ester transfer protein (CETP) in the pathways of Fat digestion and absorption and Cholesterol metabolism pathway (Figure 4a,b). These genes and their associated pathways are posited to represent the primary molecular mechanisms through which FFDS exerts its therapeutic effects on SCHD [according to the TCMSP database, hexanal in FFDS targets CD36; (2S)-2-aminosuccinic acid targets FABPpm; oleic acid and caprylic acids target L-FABP; oleic acid also targets CETP and LPL; and myristic acid targets LCAT].

## Discussion

Published meta-analyses of clinical studies have demonstrated that incorporating FFDS into the treatment regimen for CHD can enhance patient outcomes related to MACE, TG, LDL, HDL, and angina (13, 18). Consistent with these research trends, our study also identified a beneficial effect of FFDS in reducing the incidence of angina in patients with SCHD. However, regarding blood lipid profiles, our study indicated that FFDS significantly reduces TG levels, while it does not exhibit a notable effect on other lipid parameters. In these meta-analyses, notably, the observed discrepancies in the effects on blood lipids, such as LDL and TC, were accompanied by substantial heterogeneity (*I^2^*), which the authors did not further investigate. Upon reviewing the original studies included in the meta-analyses, we hypothesized that the observed heterogeneity might be attributed to variations in study population sample sizes, the criteria employed to define CHD, and the duration of medication administration. Furthermore, our study demonstrated that the use of FFDS did not result in a further reduction of MACE in patients with SCHD, which contrasts with findings from previous research (18, 19). It is noteworthy that the follow-up period in prior studies was six months (19), whereas our study reported a *P* value of 0.08 with a follow-up duration of three months. This leads us to believe that extending the follow-up period may yield an unexpected result. In summary, contemporary research indicates that FFDS exhibits a therapeutic effect in patients with SCHD, which is evidently associated with its ability to reduce serum TG levels.

TG are predominantly located in adipose tissue, whereas circulating TG are primarily present in TG-rich lipoproteins, including chylomicrons, very-low-density lipoproteins (VLDL), and their remnants (20). These lipoproteins have been identified as significant contributors to the development of atherosclerosis, which serves as the primary pathological foundation for CHD (21). Elevated plasma TG levels may arise from the dietary intake of exogenous TG, as well as from the hepatic synthesis and secretion of TG-rich VLDL, with the former being the principal contributing factor (22).

Exogenous TG are subjected to hydrolysis by bile acids and pancreatic lipase, followed by absorption in the small intestine (23). As illustrated in Figure 4a, dietary fats are hydrolyzed by bile acids and pancreatic lipase into lysophosphatidic acid, 2-monoacylglycerol, and fatty acids. These smaller molecules are absorbed into the cytoplasm through the brush border membrane of the epithelial cells lining the small intestine and are subsequently stored in the endoplasmic reticulum. The prechylomicrons are subsequently assembled and transported through the Golgi apparatus, where they mature into chylomicrons. These chylomicrons are then secreted into lymphoid tissue through the process of exocytosis. From there, they are conveyed into the bloodstream via lymphatic vessels, ultimately entering the circulatory system through the thoracic duct. Within the bloodstream, chylomicrons facilitate the release of TG, cholesterol esters, and phospholipids, contributing to the formation of HDL, VLDL, IDL, and LDL (Figure 4b) (24).

Functionally, extracellular fatty acids can interact with CD36 on the cell brush border-membrane, the latter facilitating their entry into the cell and subsequent conversion of Coenzyme A to Acyl coenzyme A within the smooth endoplasmic reticulum (25). Acyl coenzyme A plays a crucial role in the conversion of Diacylglycerol to TG (26). Similarly, FABPpm also exists in the brush border-membrane of small intestinal epithelial cells and serves as another carrier for the transport of extracellular fatty acids into cells (27). Furthermore, L-FABP facilitates the transport of absorbed free fatty acids to the endoplasmic reticulum, thereby initiating the early stages of chylomicron formation (28). Collectively, CD36, FABPpm, and L-FABP are integral to the absorption and transport of intestinal free fatty acids, contributing to the subsequent formation and elevation of TG. Interestingly, our study suggests that hexanal present in FFDS may target CD36, while (2S)-2-aminosuccinic acid may target FABPpm, and both oleic acid and caprylic acid may target L-FABP. These may be the key mechanisms for FFDS to reduce TG level.

In the subsequent phase, chylomicrons entering the circulation facilitate the generation of nascent HDL through the release of TG (Figure 4b). The LCAT catalyzes the transfer of the C2 unsaturated fatty acid from lecithin within nascent HDL to free cholesterol, thereby producing lysolecithin and cholesteryl ester (29). This process results in the production of cholesteryl ester as a direct outcome of LCAT catalysis. It is estimated that approximately 70% to 80% of cholesteryl ester in plasma is synthesized by LCAT (30). Furthermore, cholesteryl ester can be converted into TG under the regulation of CETP (31, 32). Additionally, Additionally, TG can be transformed into cholesterol esters through CETP activity (32). Therefore, LCAT and CETP serve as significant regulators of circulating TG levels. Notably, our research indicated that FFDS containing oleic acid and myristic acid may influence CETP and LCAT, respectively, potentially representing an additional molecular mechanism through which FFDS modulates TG levels.

In addition to improving the progression of stable CHD by reducing circulating TG levels, FFDS appears to be involved in disease regulation through other mechanisms. LPL hydrolyzes TG in chylomicrons, resulting in the formation of remnant particles that can be recognized and taken up by liver cells (22). Normally, chylomicrons derived from food cannot directly penetrate the subendothelium of blood vessels due to its large volume, but its remnant particles, similar to LDL, can infiltrate the subendothelium, triggering atherosclerosis (22). In addition, upon degradation of TG by LPL, free fatty acids are released, leading to local inflammation and the formation of foam cells as monocytes phagocytose lipids (Figure 4b) (22, 33). Furthermore, LPL continue to hydrolyze TG in VLDL and LDL, forming smaller and denser LDL particles compared to normal (34). These small, dense LDL particles are metabolized less efficiently by the liver, leading to their prolonged presence in the bloodstream and an increased likelihood of deposition on the vascular wall, thereby promoting the development of atherosclerotic lesions (33, 35). Our study indicated that the oleic acid present in FFDS may interact with LPL, potentially serving as a complementary mechanism for FFDS in the treatment of stable SCHD.

Despite conducting clinical and preliminary mechanistic studies to evaluate the efficacy of FFDS in treating SCHD, certain limitations impede a comprehensive interpretation of the results. Firstly, it is important to acknowledge that this study is a single-center, open-label, non-double-blind RCT with a limited sample size, which undermines the robustness of the clinical evidence. Furthermore, our study was restricted to predicting the potential mechanism of action using network pharmacology. Although we conducted further investigation into the findings of network pharmacology using lipid metabolome analysis, additional research is required to validate the absorption of FFDS monomers and to elucidate their specific mechanisms of action with their respective targets. Additionally, the lipidomics analysis was limited to 30 venous blood samples from the FFDS group and 30 from the control group. This limited sample size further undermines the reliability of the lipidomics results and the subsequent predictions regarding the molecular mechanisms of FFDS in treating SCHD. However, despite these limitations, this study provides partial evidence and insights into the potential mechanisms of FFDS in treating SCHD, offering valuable guidance for future research. Subsequent investigations could involve clinical studies with higher levels of evidence, such as multi-center, large-sample, double-blind RCTs, incorporating additional outcome measures and extending the follow-up period to comprehensively evaluate the effects of FFDS on SCHD. Furthermore, the molecular basis, including the absorption of FFDS's active ingredients into the bloodstream and their distribution concentration in specific tissues such as intestinal epithelial cells, will be explored. This will also be achieved through experiments focusing on molecular-protein interaction mechanisms to investigate the interactions between compounds like hexanal and (2S)-2-aminosuccinic acid in FFDS, and targets such as FABPpm, L-FABP, and CETP.

## Conclusion

FFDS has demonstrated its potential in reducing circulating TG levels and angina frequency in patients with SCHD, which may be due to the active ingredients of FFDS acting on LPL, CD36, FABPpm, L-FABP, LCAT, and CEPT.

## Data Availability

The original contributions presented in the study are publicly available. This data can be found here: http://www.ebi.ac.uk/metabolights/MTBLS12172 (36).
