# Supplementary material for: Therapeutic effect and potential mechanism of Fufang Danshen dripping pills for stable coronary heart disease: a randomized controlled trial
Source: Front Cardiovasc Med. 2025 Jan 29;12:1506917. doi: 10.3389/fcvm.2025.1506917 (PMC11813928; doi:10.3389/fcvm.2025.1506917)
Supplement: Supplementary file 1 [file Datasheet1.pdf]

Contents

Supplementary Table 1. 236 chemical components contained in FFDS..2
Supplementary Table 2. 201 symbols of FFDS targets 8
Supplementary Table 3. 1,576 therapeutic targets for CHD and 471 therapeutic targets for hyperlipidemia..8
Supplementary Table 4. 28 therapeutic targets that FFDS may act upon in patients with SCHD 16

Supplementary Table 1. 236 chemical components contained in FFDS.

protocatechuic acid
5-isopropyl-2-methylbicyclo[3.1.0]hex-2-ene
[(3R)-3,7-dimethylocta-1,6-dien-3-yl] acetate
Cymol
NERYLACETATE
EIC
protocatechualdehyde
1,2,5,6-tetrahydrotanshinone
beta-Chamigrene
Poriferasterol
poriferast-5-en-3beta-ol
D-Camphene
isoimperatorin
(R)-linalool
cyanidol
Moslene
1H-Cycloprop(e)azulen-7-ol, decahydro-1,1,7-trimethyl-4-methylene-, (1aR-(1aalpha,4aalpha,7beta,7abeta,7balpha))-
sugiol
caffeic acid
(-)-beta-Phellandrene
oleanolic acid
Dehydrotanshinone II A
VIV
Baicalin
[(1S)-endo]-(-)-Borneol
succinic acid
beta-caryophyllene
L-Serin
Threonin
PHA
LPG
tigogenin
Physcion
TMH
GLY
ursolic acid
Gulutamine
L-
(-)-Epicedrol
Leucinum
h-Met-h

L-Lysin<br>DTY<br>digallate<br>isoferulic acid<br>luteolin<br>Prolinum<br>8-isopropylidene-1,5-dimethylcyclodeca-1,5-diene<br>ASI<br>L-Valin<br>oleic acid<br>L-Ile<br>palmitic acid<br>(E)-3-(3-hydroxy-4,5-dimethoxy-phenyl)acrylic acid<br>5,6-dihydroxy-7-isopropyl-1,1-dimethyl-2,3-dihydrophenanthren-4-one<br>1,2-DT-Quinone<br>Dehydromiltirone<br>1-ketoisocryptotanshinone<br>2-isopropyl-8-methylphenanthrene-3,4-dione<br>3α-hydroxytanshinone II a<br>3beta-Hydroxytanshinone IIA<br>(1R,4R,5S)-1-isopropyl-4-methyl-4-bicyclo[3.1.0]hexanol<br>(E)-3-[2-(3,4-dihydroxyphenyl)-7-hydroxy-benzofuran-4-yl]acrylic acid<br>4-methylenemiltirone<br>2-(4-hydroxy-3-methoxyphenyl)-5-(3-hydroxypropyl)-7-methoxy-3-benzofurancarboxaldehyde<br>6-o-syringyl-8-o-acetyl shanzhiside methyl ester_qt<br>7-oxoroyleanone2<br>(4bS,8aS,10S)-10-hydroxy-2-isopropyl-4b,8,8-trimethyl-5,6,7,8a,9,10-hexahydrophenanthrene-3,4-dione<br>formyltanshinone<br>3-beta-Hydroxymethyllenetanshiquinone<br>Methylenetanshinquinone<br>przewalskin a<br>przewalskin b<br>przewalskin c<br>przewalskin d<br>Przewaquinone A<br>Przewaquinone B<br>przewaquinone c

(6S,7R)-6,7-dihydroxy-1,6-dimethyl-8,9-dihydro-7H-naphtho[8,7-g]benzofuran-10,11-dione
przewaquinone f
(2S)-3-(3,4-dihydroxyphenyl)-2-hydroxypropanoic acid
saloilenone
sclareol
tanshinaldehyde
Tanshinol A
Danshenol B
Danshenol A
Z-8-Hexadecen-1-ol acetate
Salvilenone
carnosol
cryptotanshinone
dan-shexinkum a
dan-shexinkum b
dan-shexinkum d
danshenspiroketallactone
danshenspiroketallactoneii
deoxyneocryptotanshinone
dihydroisotanshinone Ⅰ
Istidina
dihydrotanshinlactone
dihydrotanshinone Ⅰ
diisopro-penyl methyl vinyl cyclohexane2
epidanshenspiroketallactone
ethyl lithospermate
C09092
isocryptotanshi-none
isosalvianolic acid c
isotanshinone iib
Isotanshinone II
Isotanshinone I
lithospermic acid
manool
methylrosmarinate
methyltanshinonate
miltionone Ⅰ
miltionone Ⅱ
miltipolone
Miltirone
neocryptotanshinone ii
neocryptotanshinone
przewalskin
1-methyl-8,9-dihydro-7H-naphtho[5,6-g]benzofuran-6,10,11-trione
potassium salvianolate d

prolithospermic acid
(2S,3S)-2-(3,4-dihydroxyphenyl)-7-hydroxy-4-[(E)-3-hydroxy-3-oxoprop-1-enyl]-2,3-dihydrobenzofuran-3-carboxylic acid
(2R)-3-(3,4-dihydroxyphenyl)-2-[(Z)-3-(3,4-dihydroxyphenyl)acryloyl]oxy-propionic acid
salviacoccin
danshensu
salvianic acid c
salvianolic acid a
salvianolic acid c
salvianolic acid d
salvianolic acid g
salvianolic acid j
salvilenone I
salviolone
methyl (1S,4aS,5R,7S,7aS)-5,7-dihydroxy-7-methyl-1-[(2S,3R,4S,5S,6R)-3,4,5-trihydroxy-6-(hydroxymethyl)oxan-2-yl]oxy-4a,5,6,7a-tetrahydro-1H-cyclopenta[d]pyran-4-carboxylate
shanzhiside methyl ester_qt
(6S)-6-hydroxy-1-methyl-6-methylol-8,9-dihydro-7H-naphtho[8,7-g]benzofuran-10,11-quinone
Tanshindiol B
Przewaquinone E
Tanshilactone
tanshinone iia
(6S)-6-(hydroxymethyl)-1,6-dimethyl-8,9-dihydro-7H-naphtho[8,7-g]benzofuran-10,11-dione
tanshinone VI
tanshinone i
Spirostan-3-ol, (3beta,5alpha,25S)-
β-cadinol
apigenin
stearic acid
hexadecane
luteolin-7-o-glucoside
(R)-p-Menth-1-en-4-ol
Germacrene D
(1R,4S,4aR,8aR)-4-isopropyl-1,6-dimethyl-3,4,4a,7,8,8a-hexahydro-2H-naphthalen-1-ol
1,8-cineole
(+/-)-Isoborneol

Myrcene
(S)-(+)-alpha-Phellandrene
ZINC00968101
Hemo-sol
()-alpha-Terpineol
()-Borneol
CHEBI:7
beta-Humulene
(-)-Epoxycaryophyllene
beta-elemene
Neryl acetate
(S)-camphor
Isosafrole
α-pinenepinene
β-terpineol
()-beta-Pinene
(5S)-1-isopropyl-4-methylbicyclo[3.1.0]hex-3-ene
LINALOOL (D)
PEL
2-ACETYLPYRROLE
(+)-Ledol
myristic acid
Mandenol
Daturic acid
Hypnon
alpha-Guaiene
zoomaric acid
DFV
Cadalin
ZINC02169908
hexanoic acid
PTL
farnesol
3691-11-0
Picein
Piceol
butylated hydroxytoluene
Diop
caprylic acid
lauric acid
Sitogluside
beta-sitosterol
2-Coumarate
5-METHYLFURFURAL
MEHQ
Stigmasterol
2-octanone
ginsenoside rh2

1-Hydroxycumene
(-)-alpha-cedrene
alloaromadedrene
hexanal
[(1R)-1-methoxyethyl]benzene
Cyclooctadiene
1,2-DIHYDRO-1,5,8-TRIMETHYLNAPHTHALENE
1,4,6-trimethyl-1,2,3,4-tetralin
(1R,2S)-1-ethyl-2-methylcyclopropane
1-methyl-5-isopropenyl cyclohexene
DICHLOROANILINE
2,6-dimethyl-cyclohexanol
(2E)-3-ethylpenta-2,4-dien-1-ol
(9Z,12E)-octadeca-9,12-dienoic acid methyl ester
ZINC01532096
Butylcyclobutane
isopulegone
NaPst
10Z,13Z-nonadecadienoic acid
panaxatriol
panaxydol
NSC692928
NSC 308879
WLN: QR DG
α-cyperene
α-copaene
(5S)-5-ethyloxolan-2-one
methyl palmitate
Hepanal
Ethylpalmitate
quercetin

Supplementary Table 2. 201 symbols of FFDS targets

ACHE GABRA1 RELA EGFR VEGFA CCND1 BCL2 FOS EIF6 CASP9 PLAU RB1 IL6 AHSA1 CASP3 TP63 ELK1 NFKBIA POR CASP8 RAF1 PRKCA HIF1A RUNX1T1 ERBB2 PPARG ACACA CYP3A4 CAV1 MYC CYP1A1 ICAM1 SELEVCAM1 PTGER3 BIRC5 DUOX2 NOS3 HSPB1 MGAM CYP1B1 CCNB1 ALOX5 GSTP1 NFE2L2 NQO1 PARP1 AHR PSMD3 SLC2A4 COL3A1 DCAF5 NR1I3 CHEK2 HSF1 CRP RUNX2 RASSF1 CTSD IGFBP3 IGF2 IRF1 ERBB3 PON1 DIO1 NPEPPS HK2 RASA1 GSTM1 GSTM2

Supplementary Table 3. 1,576 therapeutic targets for CHD and 471 therapeutic targets for hyperlipidemia

1,576 therapeutic targets for CHD	471 therapeutic targets for hyperlipidemia
APOE APOB LDLR LPA MTHFR PLA2G7 HNF1A TCF7L2 ZC3HC1 ABCG5 DNAH11 CX3CR1 ACE MRAS LPL MMP3 PON1 CD36 IRS1 VKORC1 CCDC92 ESR2 PLAU EDN1 MIR17HG MMP9 MACO1 CCL2 CCR5 CD40LG MIF CDKN2B-AS1 ABCC9 CETP ABCA1 ALDH2 PHACTR1 PCSK9 ABO KCNJ8 LIPC LOX ALOX5 HMGCR IL6R TCF21 ADAMTS7 PLPP3 SCARB1 PECAM1 LIPA FADS1 SLC22A3 LPAL2 NR1H3 COL4A2 MIA3 JCAD FADS2 SH2B3 AR SMAD3 ADTRP ZPR1 CYP17A1 CELSR2 ABCG8 SMARCA4 FTO PROCR GALNT2 MTHFD1L GCKR ZFHX3 UBE2Z BRAP KCTD10 FLT1 ANKS1A KCNJ11 ATP2B1 CNNM2 WDR12 SVEP1 HHIPL1 ALDH1A2 ZSCAN16-AS1 LINC00240 SLC17A4 ADAMTS9-AS2 TARID LINC01016 MYZAP CERT1 RAD50 LIPC-AS1 TSBP1-AS1 LINC01339 NUTF2 HCG9 CDKN2A CDKN2B BTN3A3 BTN2A2 TOMM40 LINC02463 MECOM-AS1 IGF2BP2 SLC17A3 CFTR HCP5 TRAFD1 MORF4L1 FILIP1L PLCD3 KIF12 GLCCI1 CHRNA3 CHRNB4 KLHL29 ZBED9 APOA5 ZNF831 COL11A2 MUCL3 ASZ1 CR1L CRP PLEKHA7 GLT1D1 CSK GCOM1	LDLR PPARG PPARA APOE LPL CETP APOB ANGPTL4 LEPR KLF14 PLTP TOMM40 NECTIN2 HP BCL3 ABCG5 AHR DNAH11 ATP7B MACO1 VNN1 NR5A2 PEX11A NEIL1 TLR2 FABP2 TNF CRP PCSK9 IL6 APOA5 GCG ADIPOQ APOC3 APOA1 LPA LEP MTTP ACE GCKR LMNA SERPINE1 ANGPTL8 CD36 ANGPTL3 SCD MTHFR ABCA1 ADRB3 RBP4 LIPA IL10 IRS1 TLR4 EHMT1 DPP4 GLP1R NOS3 ALB PPARD RETN FGF21 CNR1 TCF7L2 USF1 CAV1 LIPE MYLIP NR3C1 LIPC REN GPT COG2 GH1 ENHO GCK PTPN1 CNBP CLOCK ACLY ZGLP1 XPR1 MTOR VCAM1 CPB2 SLC17A5 ALPP ATRNL1 ATHS MIR122 PDLIM3 CCL27 SCARB1 IL1B NR1H3 IFNG HMGCR SOCS3 AR DGAT2 NAT10 FADS1 GOT2 ASRGL1 APOC2 ESR1 ADRB2 RARRES2 APOM PON1 HNF1A FABP4 SREBF1 SLPI AGT SORT1 BUD13 LCAT CHDH ST3GAL4 GGTLC5P STAP2 KRT16 CXCL12 GOLGA6A RXRA ACE2 ATXN2 IL18 RAPSN CCL2 PTGDS IL6R PNPLA2 IL17A NR1I2 MC4R SOD1 SOAT2 MTRR GGT2 MUSK ABCB1 ENPP1 GGTLC3 PCSK1 NPY TPD52 NPY5R

CST3 NKX2-5 HJV CYBA	INSIG2 MLXIPL TNFRSF11B PIK3R1
CYP2C19 TTC39B CYP21A2 SLC30A8	GGTLC4P FLAD1 RCBTB1 SOD2
AGER DNM2 AGT AGTR1 ESR1	LRP5 FTO LINC01194 LTA BRD2
MECOM ALB F2 CABCOCO1 KIF6	VDR POMC TGFB1 PLG PKD1
JAZF1 FHIT RPH3A SMG6 CUX2	TP53 TXNIP HGF ACACA FOXC2
SIK3 SIRT1 ABL1 TTC41P	AHSG SLCO1B1 FOXO1 BTN2A1
GABBR1 GABRB1 GATA4 HLA-DPA3	AGTR1 CDKN2A FLNA FSHMD1A
HPGDS ANGPTL3 HECTD4	SIRT1 ADACST3 CYP7A1 HPGDS
HNF1A-AS1 GSTM1 GSTT1 GRHL1	EPO PLIN2 NPC1L1 GCGR APOA2
HFE ZNRD1 HLA-A HMOX1 HNF4A	ESR2 GGT1 ALDH2 AKT2
HP APOA1 APOA4 ICAM1 APOC3	HNF4A GPR119 THBS1 SHC1
HLA-V IL1B IL1RN IL6 CXCL8	SHBG CYP2B6 SPARC NOD2
IL10 IL17A IL18 ITGB3 ITPK1	SRI CIDEC TIMP4 SPP1 CYBB
ITPR3 LHFPL3 KCNQ1 UBE2Q2P1	CR1 TGFBR2 CTSD PLA2G7
SFTA2 COL11A2P1 LEP TOMM5	CRABP2 SLC15A1 CTSG CPT2
LTA MIR126 MIR146A HCG18 HCG17	NRG4 ADAM17 SLC5A2 SLC2A4
MEF2A MMP1 MMP2 MPO	CYP2B7P SDC1 CYP2J2 SPRED2
CATSPER2P1 MTR MTRR MYL2	ZBTB7C PTX3 MIB1 MIR509-1
PPP1R12B NFKB1 NOS3	MIR518C PTGS2 AKT1 BAD BAAT
NOTCH2 OLR1 TNFRSF11B	CHPT1 MAP2K1 MAPK7 SULF2
SERPINE1 SERPINA5 ANGPTL4	EDN1 UBL5 CCND1 SCP2
POLK GALNT7 PLA2G1B PLA2G2A	BGN CYP11B2 MSMO1 RENBP ACSM3
PLCG1 SEMA5B PON2 PPARA	AWAT1 BDNF RORA ROCK1
PPARG CDKAL1 DUS2 ULK4	DBP DECR1 SLC30A8 BCL2A1 F2
CHDH STK32B MAML3 ZCCHC8	NR1H4 TRIM47 PRG4 SELENBP1
CARMIL1 POLR3B RNF130 BCAP29	SIGLEC12 ZPR1 SGPL1
RETN SLC12A9 PTGS2 SUGP1	ANGPTL7 APLN TRIB1 FSTL3
REN BDNF SORT1 ATXN2	MBTPS1 LOC102723407 DGAT1
CXCL12 THADA PCIF1 GPSM3	KCNK5 CAV3 LOC102724197
SELE SELP SLC39A8 DPEP2 DPEP3	TIMD4 GGTLC1 RBM8A MAFB
ZSCAN31 SHBG P2RY12 SKIV2L	OSBPL2 BMS1 PIEZO1 RAPGEF5
SLC22A1 BTD TGFB1 THBD TLR4	CDKN2B-AS1 TBPL1 ALL2
TSPAN8 TNF TP53BP1 TRIO	MIR3659 ROCK2 FADS2 LIPG ABCB6
DHFRP2 VDR VEGFA VWF	KL DNM1L PIAS1 UBASH3B
ZNF165 SLC30A3 CACNB2 CAD	XDH XBP1 VEGFA CHI3L1
TMEM231 NAA25 ACAD10	WDHD1 UCP3 MGLL OSBPL10
ZNRD1ASP DIAPH3 PLA2G6 NLRC5	TRPS1 TRPC5 C3 CCR5
CMAHP CMSS1 SRRM4 FNDC1	COL4A1 TNNT1 SLCO6A1 COX8A
FRMD5 DNAJC5B BAZ1B	MIR34A ZNF202 AOPEP NCOA2
CHRDL1 FAM114A1 CD14 KL	NCOA3 CAMP MKKS LPAL2
ADIPOQ LIPG KIAA0319 F7 FGB	RN7SL263P CALCR HDAC11
ALOX5AP NPC1L1 CFH IL1A	SLC17A3 OGFOD3 SLC27A5 RHBDF2
MIR155 PCYT1A PLG TBX20 GDF15	CHPF RIPK3 CAD CPT1A FAAH
TRIB1 CITED2 CYP1A1 DECR1 F5	MMP9 LTF LTBR ARNT
OR10A4 HSPD1 IFNG ITGA2B	GPR162 GLRX LRP2 GNB3
KDR MIR499A MOK CCL5 TBX5	IFNL3 LOX OR10A4 GPX1 GRN
CCR2 USF1 NLRP3 CYP2J2 F3	FLVCR1 KLF15 LGALS1 ANPEP
FBN1 MMRN1 GJA4 NR3C1	IL37 MIR130A MMP3 MME NR3C2
HMGB1 MIR21 MIR223 NPY ABCB1	MKI67 DNAJB9 NOX1 ARSA

APOM CXCL16 TFPI CD40<br>C20orf181 CERNA3 ADD1 APLNR<br>FABP4 FLNA ALOX15 GNB3<br>GP1BA GPX1 ISL1 ABCC6 KNG1<br>LEPR ARSD MPI MYH6<br>NOTCH1 HSPA14 PON3 CCHCR1<br>PPARD BCHE ACTB S100A12<br>SELENBP1 KIF28P NAMPT<br>MTCO2P12 HPSE KIF2C<br>COL4A1 SLCO6A1 ADRA2B PLB1<br>ADRB2 FCGR3A ZFPM2 PLA2G15<br>GATA6 FGF21 GC AMPD1<br>ANGPT2 HIF1A IGF1 GSTK1 LRP6<br>COX2 MUSK ATM FOXP3<br>MLXIPL PIK3CG PPIAAVP ACSS2<br>PTX3 RAPSN ACE2 RBP4<br>SELLSELPLG SOD1 TNFRSF1B<br>TP53 VPS51 YWHAZ FLAD1<br>ACCS CAT CBS HAND1 CBSL<br>PPARGC1A CHI3L1 CPB2 ADRB3<br>CYP2B6 CYP2C8 CYP11B2 CYP19A1<br>DBP EGFR EGR1 EPHX2 FABP2<br>FCGR3B FSHMD1A POU2F3 COPD<br>GATA2 GDF1 GLP1R GSTM2<br>GUCY1A1 HGF HLA-DRB1<br>HSPA1A APOC1 IFNA1 IFNA13<br>FAS IL16 ENHO LCN2 LGALS2<br>LGALS3 MIR17 MEFV MGP<br>NR3C2 MTTP GP6 PIK3CA PIK3CB<br>PIK3CD PITX2 UGT1A1 PPBP<br>PSMA6 PTH HPSE2 S100B BHMT<br>SREBF1 TBX1 TNFSF4 UCP2<br>XRCC1 ZIC3CA2 SLC25A20<br>CRELD1 FSD1 FSD1L PLA2G10<br>PSRC1 APLN ABCG1 HDAC9<br>ADAADAR KLF2 CLU ADH1C<br>CNR1 CYP3A4 CYP4A11CYP7A1<br>DPP4 DSCAM RCAN1 ECE1<br>AHRAHSG EPO EPRS1 ETS2<br>FABP3 FCGR2A FGFR4 TBC1D9<br>FN1 ANGPTL2 HEY2 DDAH2<br>SMUG1 IL37 C5AR2 GPR162 GLUL<br>BTBD8 GPT ANXA2 HLA-DQB1<br>HSPA1B HTC2 APOA2 TNC<br>C1QTNF9 NANOS3 PEAR1 LCAT<br>LMNA MIRLET7C MIR143<br>MIR145 MIR149 MIR206 MIR23A<br>MIR34A ARSA MC4R MEF2C<br>MMP12 NFATC1 NFE2L2 NODAL	SMAD2 MIR96 NAAA MIR33A<br>MIR27B MIR22 MIR195 MIR148A<br>MIR146AGSK3B LECT2 HLA-A<br>CXCL8 HMGB1 HMOX1 IL4 FOXA2<br>IL1AIGFALS HSD11B1 HSD17B13<br>HSPG2 IFNA13 HTR2C IFNA1<br>IDH2 ID2 IRF2BP2 ANXA6 REPIN1<br>LCN2 APOA4 C1QL3<br>SERPINA13P UBIAD1 ANXA1 KCNH1<br>MIA3 GSTK1 JAK2 ABCC6<br>HADHA INSR INPPL1 INS HCAR2<br>TMEM126B PODXL BCO1 SARM1<br>TBC1D9 PLCG2 TM6SF2 PLAT<br>GGA3 MPRIP ACP1 PECAM1<br>WWOX ENPP2 PASK IL23A<br>SIRT6 TREM1 SMG1 ACOT13<br>PRKAB1 PRKAA2 FABP1 PRKAA1<br>PREP PTK2B FASN FBN2<br>TMEM132A DYM PPIAMRAS<br>SCAP FAIM2 CCHCR1 SCLY<br>DCDC2 GALNT2 LDLRAP1NM NF2<br>NDUFS4 PTPN22 GATA3 NBN<br>MYLK MYD88 MVK DAG1<br>MTRBSCL2 MT1B MSRA CCN3<br>G6PC PDC FLT1PCOS1 IPO11<br>FMO3 SIK3 ABHD5 SIRT3<br>SERPINA5 PAX5 PAPPA NOX4<br>PAEP OLR1 NUCB2 NRF1<br>MPO

NOS2 P2RY1 PAPPA ADIPOR1<br>ENPP1 PLTP RNLS PROC<br>JPH3 PTGS1 HAMP BAX ACTA2<br>RARRES2 BCL2 RFC1 S100A9<br>NOD2 BMP2 SLC5A2 SOD2<br>SPP1 SREBF2 TBX2 TBX3<br>TCN2 ACTC1 TERC NR2F2<br>TMSB4X TNFRSF1A TNNI3 C3<br>POTEF AVSD1 EHMT1 KMT2D<br>CASP1 CASQ2 KALRN F2RL3<br>IL33 NEXN CD28 HAND2 CD59<br>NOS1AP NAT2 HLP ABCB6 PGR-<br>AS1 EBI3 TP53COR1 CFDP1 SLCO1B1<br>RN7SL263P CES1 NES UTS2<br>CHD2 ADAMTS13 ATXN2L H3P10<br>TMEM170A RBM45 COMT ADM<br>COX8A KLF14 CRH CSF3 IL23R<br>CTSS ADRB1 DAB2IP CYP1A2<br>CYP2C9 CYP2E1 CYP3A5 DEFA1<br>DHCR7 NQO1 DNTT AGTR2<br>EDNRA EGR3 DAND5 EPAS1<br>EPHX1 ERCC1 ERCC2 ETS1<br>F2R F10 F11 F13A1 FBN2 CD93<br>FOXO3 TAB2 FLOT2 DDAH1<br>FXN IL27 GABPA GCA POC1A<br>PLA2G2D GCK GFAP GJA5<br>GCLC ANGPT1 SETD2 GSTP1<br>APC APEX1 HSD11B1 HSPA2<br>HSPA4 BRINP3 IGF2 IGFBP3 CCN1<br>IL2 IL4 IL5 IL15 INS INSIG1<br>MALAT1 ARG1 ACAT2 LAMP2<br>CIMT MIR122 MIR130A MIR142<br>MIR150 MIR210 MIR22 MIR31<br>MIR34B MIR93 SMAD2 MBL2<br>MMP7 MMP8 ASGR1 MMP13<br>COX1 MVK NCAM1 NFKBIA<br>NHS NNMT NOS1 NPC1 NPPC<br>ACR MIR378A MIR423 OGG1<br>SERPINB2 IRX4 FCMTE1 INSIG2<br>PCSK1 PDE4D SERPINF1 PGF<br>ARL15 FIGN QRSL1 FCMTE2<br>MESP1 MAPK3 BTNL2 SLC2A9<br>B2M STARD7 PTEN MRTFA<br>HOMEZ TRIB3 MYL7 BCL2A1<br>RENBP RFX1 GAS5 ROS1<br>RXRA S100A1 SCD BGLAP SCN5A<br>CCL21 CXCL5 CX3CL1 SFRP4<br>GMCL1 GMCL2 SHH SI AIDA<br>AGXT2 WNK1 BMP4 SLC6A4	

BMPR1A BMPR2 SOAT1 BRCA1<br>ST2 STAT3 TCP1 TFAP2B<br>TGFBR2 THBS1 THBS2 TIMP1<br>TLR2 TNNT1 UBC VCAM1 VEGFC<br>VIM BEST1 CXCR4 CUBN SP6<br>SOX7 BUD13 FCN3 CAV1<br>SOCS1 IRS2 TNFSF14 TNFRSF6B<br>TNFRSF11A PROM1 NR1I2 MGAM<br>SOCS3 SLC33A1 KLF4 NTN1<br>BCAR1 CLOCK CD69 PCLAF<br>MVP SCO2 CDH2 AKT3<br>HOTAIR ZGLP1 MIR873 MIR365A<br>KLLN AFA1 MIR1233-1<br>HOTTIP PARP2 MIR4271 MIR2909<br>MIR3664 LINC01672 COMMD3-BMI1<br>MICOS10-NBL1 MIR4513 IL18BP<br>HIF1A-AS2 COX10-AS1 IFNG-AS1<br>CDH13 ATP6AP2 LPCAT3 SORBS3<br>RNF41 TSHZ1 ADAM10 MPHOSPH6<br>MPZL2 AKR1A1 MYL12B NOD1<br>DLC1 ADARB1 YAP1 APOA1-AS<br>EIF3M CAP1 H19-ICR CEBPE<br>THRA1/BTR ANP32B CECR CEL<br>MRPL28 ZRS ERLIN1 MYL12A TXNIP<br>POSTN LEFTY1 CXCR6 EBP CERS1<br>ARPP21 NEU3 NPFFR2 SUB1<br>LOC109280161 KCNQ1OT1 CHD1<br>CHD4 ESM1 WDR5 HNRNPUL1<br>BTN2A1 PTPRT CAPN10 WDHD1<br>CHIT1 SLC2A6 IL17F LINC02605<br>TMEM54 C1QTNF1 C1QTNF3<br>H3P9 H3P40 TNFRSF13C CKMT2<br>LRRC3B CMTM5 CYP2R1 CMA1<br>CCR7 ACKR2 DNAAF1 ADH1A<br>EARS2 ADH1B COL1A1 COL3A1<br>COL6A1 OSR1 COMP TAMM41<br>CORT MAP3K8 CP CPA3 CPE<br>CPS1 CR1 NKX2-6 DGKK CRIP1<br>ADORA3 GATA5 PARP1 CACUL1<br>CSF1 CSF2 SERPINA12 PWAR1<br>NRG4 IL34 CSRP1 LYPD4<br>PTPRVP CTF1 CTH CTLA4<br>CTNNB1 ADRA2A CTRB1 CTSB<br>CTSD ERFE CYBB CYP1B1<br>CYP2C18 CYP2D6 SLC7A13 SPAAR<br>CYP27A1 AP2A2 DARS1 C16orf82<br>DEFA3 ASXL1 DHFR DMBT1<br>DNAH8 DNMT1 JAG1 DTNA<br>EBF1 LPAR1 EFNA5 EGF RMST	

ELANE ZNF627 MARK2 AKT1<br>ERCC5 EXTL3 EZH2 F8 F9<br>F12 FAAH FABP1 DLEU7 FCAR<br>FCGR1A FCGR1B FCGR2B FCN2<br>SEMA3D FEN1 FGF2 FGG MLXIP<br>SCAP DAAM1 PALLD KDM1A<br>MON2 FOXM1 FOXO1 GPD1L<br>ABRAXAS2 PMPCA DICER1 SIRT4<br>SIRT3 FOLH1 NUP188 FOS<br>ARL2BP PDSS1 BHMT2 IL17RA<br>SPESP1 ALOX15B FRZB ALPL<br>FUT3 GAD1 LMOD1 ARIH1<br>NIPBL GALNT3 ASPM SOSTDC1<br>TAS2R50 GAPDH APPL1 TES GART<br>PTPN22 GAS6 KLHL3 GCGGCH1<br>GDF2 GREM1 GDF10 AMFR<br>HAVCR1 GGT1 AMH B4GALT1<br>GH1 GHR FETUB GJB2 FOXP1<br>B3GAT1 DKK3 PALD1 AGO2<br>PDCD4 GCLM GLI1 GLI3 GOT2<br>IFNL3 LINC00841 KANSL1<br>METRNL GPER1 TREML4 XKR6<br>SCAI GRK4 GRK5 GPX3<br>FLVCR1 KLF15 GRIK4 NXT1<br>MYLIP CTNNA3 GSR GSTA1<br>GUCA1B SENP1 GUK1 GUSB<br>PADI1 A1CF ANXA1 HABP2<br>HAL HAS2 HBG2 HDAC1 EHD3<br>HHEX ACACA HLA-DRB5 HLA-G<br>HMGB2 HMGA1 HNRNPA1 HOXA1<br>ACACB HOXC9 HPCAL1 MMAB<br>SERPINA9 HSPA5 HSPA8 HSPB1<br>HSPB2 HSPE1 HSPG2 GPIHBP1<br>HCAR2 IRF8 ID2 GOLGA6A APOC2<br>IFNB1 APOC4 IFNGR2 APOD<br>IGF1R IGFBP1 IGFBP4 IGFBP7<br>ACADS LCE1A IL1RAP FASLG IL7<br>CXCR1 CXCR2 IL12B IRF2BP2<br>IL15RA TNFRSF9 ILK AQP5 IDO1<br>ING1 CXCL10 AQP6 INSR<br>IRF3 IRF6 ITGA2 ITGAM ITPR1<br>JAK2 ACAT1 KLK1 KIF22<br>KRT8 SUMO4 C1QTNF12 C1QL3<br>LAG3 RPSA LBP LMAN1 LRP1<br>LRP2 LRPAP1 LSS LTB LTBP1<br>ARNTL MIRLET7I MIR132 MIR18A<br>MIR182 MIR184 MIR197 MIR19A<br>MIR19B1 MIR20A MIR208A MIR214<br>MIR221 MIR224 MIR23B MIR25	

MIR29C MIR30C1 MIR30C2 MIR32<br>MIR96 SMAD1 SMAD7 MAFD2<br>MAG STS MAS1 CD46<br>ADAM11 MEOX2 MET MFGE8 CXCL9<br>MAP3K11 ACHE ASD1 MDD1<br>MMP14 ASIPOR13G1 MIR367 ASPA<br>MSRA MT1B MT2A MTAP<br>MTHFD1 MYBMYBPC3 MYD88<br>SERPINC1 MYO6 NAGLU NBL1<br>NCL NDUFAB1 NDUFS2 RERE<br>NEUROD1 NPPA NPPB NPR3<br>NT5E ATP2B2 NTRK2 NTRK3<br>NUCB2 NUP98 MIR361 ATP4B<br>OGDH OPA1 ZBTB21 OPRM1<br>OSM OTC OXCT1 P4HB PCSK6<br>DUOX2 NOX4 IL22 AK3 F11R PC<br>SOST PCDH8 ASCC1 CPA4<br>CLEC1B TLR7 CDK18 TLR8<br>WNT16 PDE3B ISYNA1 PDGFA<br>SIRT6 IL23A NBAS RTRAF<br>CES1P1 SLC26A4 GHRL RTEL1<br>ADA2 PF4V1 ACP1 PFKL<br>ATP5PF SERPINA1 SERPINE2<br>PIGFPIK3C2A PKD1 PKP2<br>PLA2G5 PLAGL1 PLAT IL17D<br>HDL3 STX18 PLN PLXNA1<br>PMAIP1 DUOX1 ACP5 RIPK4<br>PNN TREM1 POR DGCR8 DLL4<br>POU5F1 TET2 PPICBCOR CASZ1<br>MTARC2 NADSYN1 RCBTB1 FBXW7<br>IMPACT MIR429 YOD1 CAMK2N1<br>PARL SRGN ITLN1 PRKAA1<br>OSGEP PRKCE KDM3A SELENOS<br>PRKCH ANGPTL8 MAPK8 CFC1<br>PRKY PROS1 AZGP1 MAP3K7CL<br>MEPE PRDM10 ACKR3 PSMD4<br>KMT5AP1 PSMD9 VANGL2 PTAFR<br>TAS2R38 AICDA MIR433 HNP1<br>AS3MT MIR146B MIR503 NLN<br>KIDINS220 XPO5 MIB1<br>RNF213 PTPN11 PTPRA PTPRC<br>PTPRN2 NECTIN1 RAC1 RAF1<br>RASGRF2RBL2 SENP2 OPN1LW<br>RELA RLN1 BACH2 CPAT1<br>RPA1 RPN1 MIR483 MIR484<br>RYR2 RYR3 S100A8 SAA1<br>SCN7A CCL17 CCL18 CCL19<br>CCL22 RNF123 HEATR6 CDH23<br>CENPK CRLF2 SELENOP KIF9	

POU5F1P3 SRSF1 SRSF2 LMBR1<br>NFKBIZ SFTPD DCLRE1CSGCD<br>SH3BGR POU5F1P4 NDST4<br>GORASP1 PDIA2 BMI1<br>C12orf43SLC2A3 GGTLC5P BMP6<br>SLC8A1 SLC9A3 SLC12A3 SLC19A1<br>SMARCA2 SIGLEC1 SNRNP70<br>SOD3 SOX9 SPARC SPG7<br>SPINK1 SRC TRIM21 SSB ST14<br>STAT5A STAT5B SULT1E1 STIM1<br>STK11 BSG SUV39H1 SYK SYT1<br>TAC1 ADAM17TADA2A TAGLN<br>KLF5 MIR421 MIR570 MIR574<br>MIR592 TCF15 MLXTERF1 TERT<br>TFR2 LEFTY2 TGM2 THBS4<br>THPO THRA TIMP2 TIMP4<br>C1QBP TLR3 ACTG1 TM7SF2<br>TNFAIP3 TPM1 TRAF3 TRAF5<br>TRAF6 C3AR1 TRPC3 TRPS1<br>TTR GGTLC3 DEFA1B GGT2<br>TNFRSF4 TYMS GGTLC4P<br>SUMO1 UCP1 SCGB1A1 UMOD<br>KDM6A VASP VLDLR WNT3<br>WNT5A WNT11 GET1 CNBP<br>MIR765 CACNA1C ZNF208<br>ZNF217 LRP8 BSND SCG2<br>METRN ST8SIA4 IRX1NOX5<br>SLC52A2 SAP130 CALCA ADIPOR2<br>SAP30L GHSAGBL2 CALCR STN1<br>ZNF606 CALM1 PDGFD ADAM33<br>PNPLA3 CD276 CALM2 FGF23<br>COL18A1 CALM3 SETD7<br>ST6GALNAC5MIA MKKS CAMP<br>CAPG SMC1A USP9Y AXIN2<br>SESN2 CRISPLD1 ZNF484 ESYT3<br>ROPN1L TMPRSS13 SRPX ARID5B<br>SARNP SOAT2 MAP1LC3A CASR<br>KIF2B CNDP1 OFD1 AOPEP<br>MMP23BELP1 CYP4F2 APOL1<br>DOCK7 TSLP MADD KCNK5<br>VAMP8 BECN1 GALNT4 TNFSF13<br>TNFSF10 ACTN2 CREG1 IL18R1<br>CES2 CCN4 KAT2B ARHGEF7<br>EIF2B5 AP1S2 PHOX2B MBD2<br>HSPB3 ACVR1 HAP1 MCFD2<br>TBL1Y LDB2 TBX18 FCGR2C<br>SMC3 NLRP12 IL1RL1 TIMD4<br>INTS4 XPR1 NAF1 IL32 NREP<br>CD163 GLP2R MUC16 FAM189A2	

CD86 OPN4 ABCG2 QKI CD34<br>ATG5 ADAMTS4 ADAMTS1<br>TBPL1 CHD1L CD44 PRORP<br>CD74 PCDHA9 RAPGEF5TMEM94<br>BMS1 ELMO1 SRGAP3 MFN2<br>MAFB FGF19 THOC1 RCE1	

Supplementary Table 4. 28 therapeutic targets that FFDS may act upon in patients with SCHD

ACACA ALDH2 PON1 BDNF CD36 PPARG CRP AHR NR3C1 GOT2 ESR1 PPIA CTSD VEGFA IL6 NR3C2 INS FABP1 CAV1 CALCR NOS3 CETP TLR4 VCAM1 LPL LCAT AR ESR2
